# Inhibition of MEK1/2 and GSK3 (2i system) affects blastocyst quality and early differentiation of porcine parthenotes

**DOI:** 10.7717/peerj.5840

**Published:** 2019-01-07

**Authors:** Jeongwoo Kwon, Ying-Hua Li, Yu-Jin Jo, YoungJin Oh, Suk Namgoong, Nam-Hyung Kim

**Affiliations:** 1 Department of Animal Sciences, Chungbuk National University, Cheongju, Chungcheongbuk-do, Republic of Korea; 2 Department of Animal Sciences, Agricultural College, Yanbian University, Yanji, China; 3 Genetic Engineering, Cheongchungbuk-do Veterinary Service Laboratory, Cheongju, Cheongchungbuk-do, Republic of Korea

**Keywords:** Pig embryo, Early embryo development, 2i system, Pluripotency, Enter a keyword

## Abstract

Inhibition of both MEK1/2 and glycogen synthase kinase-3 (GSK3; 2i system) facilitates the maintenance of naïve stemness for embryonic stem cells in various mammalian species. However, the effect of the inhibition of the 2i system on porcine early embryogenesis is unknown. We investigated the effect of the 2i system on early embryo development, expression of pluripotency-related genes, and epigenetic modifications. Inhibition of MEK1/2 (by PD0325901) and/or GSK3 (by CHIR99021) did not alter the developmental potential of porcine parthenogenetic embryos, but improved blastocyst quality, as judged by the blastocyst cell number, diameter, and reduction in the number of apoptotic cells. The expression levels of octamer-binding transcription factor 4 and SOX2, the primary transcription factors that maintain embryonic pluripotency, were significantly increased by 2i treatments. Epigenetic modification-related gene expression was altered upon 2i treatment. The collective results indicate that the 2i system in porcine embryos improved embryo developmental potential and blastocyst quality by regulating epigenetic modifications and pluripotency-related gene expression.

## Introduction

Preimplantation development of mammalian embryos features several embryonic differentiation events that yield the fetus and placenta. The initial steps of cell lineage determination occur in the morula stage and cells differentiate as the inner cell mass (ICM) or trophectoderm (TE). At this stage, ICM cells expressing OCT3/4 in the blastocyst stimulates the development of primitive ectoderm or endoderm, whereas the CDX2-expressing TE cells become the embryonic portion of the placenta (Niwa, Miyazaki & Smith, 2000; Niwa et al., 2005). Embryonic stem cells (ESCs) can be isolated from the ICM in blastocysts prior to differentiation into the three embryonic germ cell layers (ectoderm, endoderm, and mesoderm). Therefore, maintenance of the undifferentiated status of the ICM is crucial for the establishment of ESCs.

Several signaling pathways are involved in the differentiation of naïve stem cells. Exogenous fibroblast growth factor (FGF-2) activates FGF receptors (FGFR) and stimulates the mitogen-activated protein kinase (MAPK) pathway (Dvorak et al., 2005), whereas Wnt/β-catenin signaling pathways stimulate differentiation toward a lineage-committed cell type (Merrill, 2012). Therefore, chemical inhibition of these signaling pathways may modulate stem cell differentiation. For example, combination treatments involving the inhibition of FGFR kinases by SU5402, inhibition of the MAPK/extracellular signal-regulated kinase pathway by the MAPK kinase (MEK) inhibitor PD0325901, and stimulation of Wnt/β-catenin signaling by the glycogen synthase kinase-3 (GSK3) inhibitor CHIR99021 in the presence of leukemia inhibitory factor can suppress Embryonic stem cell (ESC) differentiation (Ying et al., 2008). Indeed, the dual inhibition of GSK3 and MEK (the 2i system) effectively promotes pluripotent cell types, and facilitates the establishment of naïve rat stem cells, which are otherwise difficult to establish (Leitch et al., 2010; Silva et al., 2008). The direct effects of these inhibitors on the signaling pathways have been characterized. Inhibition of FGF signaling is arrested in naïve epiblast cells (Kunath et al., 2007; Lanner & Rossant, 2010; Stavridis et al., 2007). Blocking of MAPK pathways, which affects downstream signaling for FGF pathways, block epiblast differentiation (Nichols et al., 2009). In addition, stabilization of β-catenin by GSK3 inhibition activates stem cell factors, such as c-MYC, ESRRB, and OCT4, which act independently or interact with other transcription factors to enhance pluripotency (Nichols et al., 1996). The mechanism underlying the effects of these inhibitors on the maintenance and differentiation of epiblasts is unknown.

The zygotic genome is epigenetically regulated by DNA demethylation and histone modifications. For example, DNA methylation at CpG dinucleotides (Howlett & Reik, 1991; Kafri et al., 1992; Monk, Boubelik & Lehnert, 1987) and histone 3 lysine 9 trimethylation (H3K9me3), have been associated with repressive heterochromatin. H3K9 methylation may be important in suppressing pluripotent-specific genes within the TE (Alder et al., 2010; Rugg-Gunn et al., 2010). In addition, histone 3 lysine 9 acetylation (H3K9ac3) has been associated with euchromatin and gene activation (Ringrose & Paro, 2004). These epigenetic changes are mediated by different proteins, including DNA methyltransferases (DNMT) (Fuks et al., 2003) and ten-eleven translocation methylcytosine dioxygenase (TET) family of proteins (Wossidlo et al., 2010, 2011). Blocking both signaling pathways involved in ESC differentiation causes DNA demethylation by activation of Tet methylcytosine dioxygenase 1 (TET1) in a JMJD2C-dependent manner and passive DNA demethylation through DNMT3A/B degradation, which is promoted by PRDM14/G9a (Sim et al., 2017).

2i treated mammalian preimplantation embryos showed increase in ICM formation and pluripotent marker overexpression. 2i-treated rat blastocysts maintain the ICM in a naïve state by blocking differentiation (Buehr et al., 2008; Li et al., 2008). Furthermore, 2i treatment of bovine embryo increases the expression levels of epiblast marker genes in ICM cells, but not in TE cell lineages (Harris, Huang & Oback, 2013). However, the inhibitory effects of the 2i system on porcine early embryogenesis is unknown. In this study, we investigated the effect of the dual inhibition of MEK and GSK3 on porcine early embryo development and ICM formation.

## Materials and Methods

### Reagents

All reagents were purchased from Sigma-Aldrich (St. Louis, MO, USA) unless stated otherwise.

### Porcine oocyte collection and in vitro maturation

Pre-estrogenic porcine ovaries were delivered in saline at temperature >30 °C from a local slaughterhouse (Farm Story Hannang, Chungwon, Chungbuk, Republic of Korea) within 1 h of collection. Aspiration of follicle fluid was performed using an 18-gauge needle with a 10 mL syringe. Cumulus-oocyte complexes (COCs) were washed with TALP-HEPES (HEPES medium supplemented with 0.1% of polyvinyl alcohol (PVA)) and were collected the COCS which densely-covered with cumulus cells. COCs were cultured in a four-well cell culture dish with in vitro maturation medium (M-199; Invitrogen, Carlsbad, CA, USA) by adding 20 ng/mL epidermal growth factor, one g/mL insulin, 75 g/mL kanamycin, 0.91 mM Na pyruvate, 0.57 mM L-cysteine, 10% (v/v) porcine follicular fluid, 0.5 μg/mL follicle stimulating hormone, and 0.5 μg/mL luteinizing hormone at 38.5 °C in an atmosphere containing 5% CO_2_ at 100% humidity.

### 2i treatment during in vitro culture and measurement of blastocyst diameter

To activate mature oocytes in vitro, MII oocytes were collected and denuded by pipetting the expanding cumulus cells surrounding the oocyte in one mg/mL hyaluronidase. The denuded oocytes were washed thrice in PBS–BSA (Dulbecco’s phosphate-buffered saline containing 0.1% bovine serum albumin) and activated using an Electro Cell Manipulator 2001 (BTX, Inc., San Diego, CA, USA) and 280 mM mannitol medium supplemented with 0.01 mM CaCl_2_ and 0.05 mM MgCl_2_. The activating electric pulse was delivered twice at 1.1 kV/cm for 60 μs each time. After activation, the embryos were cultured in PZM-5 medium, which consisted of 108 mM NaCl, 10 mM KCl, 0.40 mM MgSO_4_·7H_2_O, 0.35 mM KH_2_PO_4_, 25.07 mM NaHCO_3_, 0.2 mM sodium pyruvate, 2.00 mM Ca-(lactate)2·5H_2_O, five μg/mL gentamicin sulfate and 0.1 mg/mL BSA. Activated oocytes were placed on PZM-5 medium with 7.5 μg/mL cytochalasin B for 3 h. The embryos were washed thrice and cultured in PZM-5 medium containing dimethylsulfoxide (0.2%; control group), MEK1/2 inhibitor (PD0325901, four μM), GSK3 inhibitor (CHIR99021, 0.3 μM), or 2i inhibitor (PD0325901 four μM + CHIR99021 0.3 μM) for 144 h at 38.5 °C in an atmosphere containing 5% CO_2_. Blastocysts were collected at 144 h post-activation and images of blastocysts were taken using a Nikon TE 2000 inverted microscope in x100 (Tokyo, Japan). Mean blastocyst diameters were calculated from these data and measured using ImageJ by pixel.

### Terminal deoxynucleotidyl transferase dUTP nick end labeling assay of blastocyst quality and cell number counting

2i-treated blastocysts were washed thrice with PVA–PBS and fixed in 4% formaldehyde in PBS. After fixation, permeabilization was performed using PBS with 0.2% Triton X-100. Cell death of permeabilized blastocysts were detected using fluorescein isothiocyanate-conjugated in situ cell death detection kit transferase dUTP nick end labeling (TUNEL; Promega, Madison, WI, USA). Negative control did not add terminal deoxynucleotidyl transferase enzyme during apoptosis labeling. Positive control blastocysts were treated with an additional step for 10 min at room temperature after permeabilization with Dnase I (30,000 U/mL) in 1X Dnase buffer (10 mM CaCl_2_, six mM MgCl_2_ and 10 mM NaCl in 40 mM Tris-HCl, pH 7.9). For nuclear staining, blastocysts were incubated with 10 mg/mL bisbenzimide (Hoechst 33342; Sigma-Aldrich, St. Louis, MO, USA) for 10 min at room temperature, followed by three washes with PVA–PBS. The washed blastocysts were immediately mounted on a glass slide. Enumeration of total and apoptosis positive cells was performed using fluorescence microscopy. Cell number of blastocysts were counted by DAPI positive cells.

### Reverse transcription-polymerase chain reaction levels

Blastocysts treated for 7 days were used for extraction of mRNA using the Dynabeads mRNA Direct kit (Life Technologies AS, Oslo, Norway). Each group of blastocysts was resuspended in lysis/binding buffer and vortexed for 5 min. The extracted mRNA was washed several times and eluted using 10 mM Tris-HCl. cDNA was synthesized using the first strand cDNA synthesis kit (Legene, San Diego, CA, USA) and amplified using the WizPure qPCR Master Super Green kit (Wizbio Solutions, Seongnam, South Korea) with specific gene primers. The Primer sequences for quantitative reverse transcription-polymerase chain reaction (qRT-PCR) is shown in Table S1. The gene encoding glyceraldehyde-3-phosphate dehydrogenase was used as an internal control for normalization in all analyses.

### Immunofluorescence and confocal microscopy

Inhibitor-treated embryos were fixed with 4% paraformaldehyde in PBS containing polyvinyl alcohol (PBS–PVA) for 30 min. PBS–PVA containing 0.5% Triton X-100 was applied for 1 h to permeabilize the embryos, which were subsequently placed in a blocking solution of PBS containing 3% BSA and 0.05% Tween 20. After blocking, the embryos were incubated overnight at 4 °C with primary antibodies diluted in 1% blocking solution (PBS with 1% BSA) against octamer-binding transcription factor 4 (OCT4, sc-9081, 1:100; Santa Cruz Biotechnology, Santa Cruz, CA, USA), SRY (sex determining region Y)-box 2 (SOX2, sc-365823, 1:100; Santa Cruz Biotechnology, Santa Cruz, CA, USA). Each sample was washed thrice in washing solution (PBS with 0.05% Tween 20) for 10 min, followed by addition of the fluorophore-labeled secondary antibody (Alexa Fluor 488 or 568; 1:200; Thermo Scientific, Waltham, MA, USA) diluted in the blocking solution. The embryos were then stained with Hoechst 33342 (10 mg/mL in PVA–PBS) for 10 min. After washing thrice, the embryos were mounted on glass slides with Vectashield (94010; Vector Laboratories, Burlingame, CA, USA) and examined using a model LSM 710 META confocal laser scanning microscope (Carl Zeiss, Jena, Germany).

### Statistical analyses

For each experiment, at least three replicates were performed using >20 embryos. Statistical analyses were performed using GraphPad Prism (GraphPad Software V6.01, La Jolla, CA, USA). One-way analysis of variance, followed by Tukey’s single or multiple comparison tests were performed. Descriptive statistics are presented as mean or standard error of the mean.

## Results

### Effect of MEK/GSK3 inhibition on porcine early embryo development

**Figure 1:**
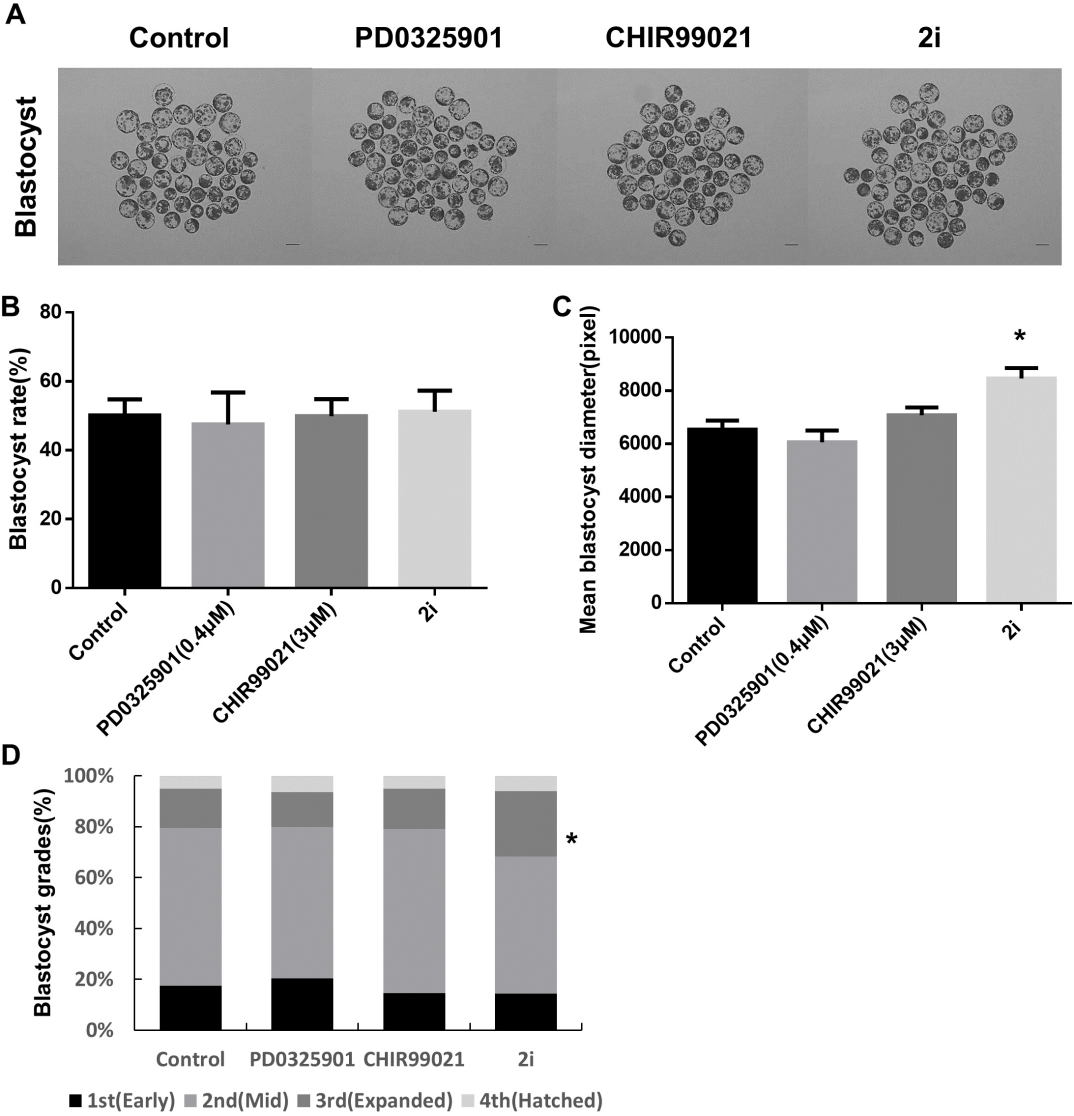
Effect of treatment with MEK/GSK3 inhibitor alone or combination on activated porcine embryos. (A) Representative microphotographs showing blastocyst formation after treatment with inhibitor PD0325901 (0.4 μM), CHIR99021 (three μM) and 2i (PD0325901 0.4 μM + CHIR99021 three μM. (B) Blastocyst rate counts in early to expanded blastocyst. (C) Mean size of blastocyst (pixels) in the surface area was analyzed using ImageJ. (D) Grade 1–4 blastocysts were categorized as early (E), mid (M), expanded (X), and hatched (H) stages on day 7. Scale bars: 200 mm. *P < 0.01.

We examined the effect of 2i treatments during porcine embryonic development. Porcine parthenogenetic embryos were treated separately or together with MEK and GSK3 inhibitors and the effect on the rate of blastocyst formation was assessed. No differences were evident between the control and 2i treatment groups (Figs. 1A and 1B; Control: 50.72% ± 2.54 vs PD0325901: 49.97% ± 5.54 vs CHIR99021: 52.52% ± 2.08 vs 2i: 53.18% ± 3.26). However, 2i treatments were associated with a significantly larger blastocyst mean diameter than the control (Fig. 1C; Control: 6,541 ± 334.3 vs PD0325901: 6,061 ± 443.6 vs CHIR99021: 7,082 ± 291.7 vs 2i: 8,463 ± 394.8). Blastocyst diameter can be used to evaluate the quality of blastocysts produced in vitro (Lagalla et al., 2015). Grading of the stages of grown blastocysts revealed that 2i-treated embryos increased the expanded blastocyst ratio (Fig. 1D), and these results revealed that 2i treatment improved blastocyst quality compared to the control.

**Figure 2:**
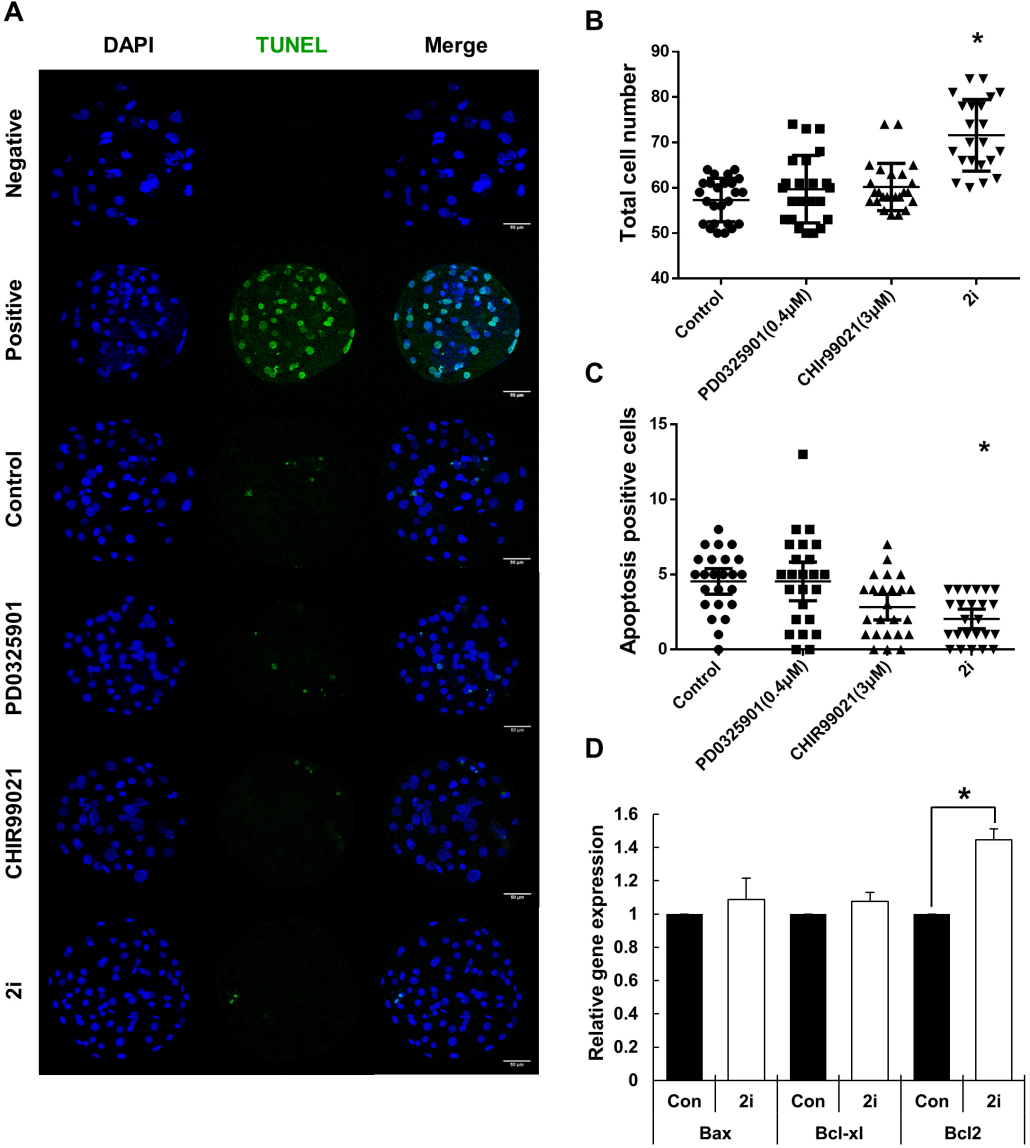
Improvement of blastocyst quality after treatment with MEK/GSK-3 inhibitor. (A) Inhibitor-treated blastocysts (Day 7) were stained for TUNEL assay for enumerating apoptosis-positive cells. Negative: Without TdT enzyme, Positive: Dnase I treated blastocyst, Control: 0.2% DMSO, PD0325901 0.4 μM, CHIR99021: three μM, 2i: PD0325901 0.4 μM + CHIR99021 three μM. (B and C) Total cell number and apoptosis positive cells were counted on day 7 after Hoechst 33342 and TUNEL staining. (D) Apoptosis-related genes expression were assessed by quantitative RT-PCR in the control and 2i-treated blastocysts. Each group consisted of >20 blastocysts. Data was normalized to GAPDH levels. Scale bars: 50 μm. *P < 0.01.

To further investigate blastocyst quality following 2i treatment, cell numbers and the prevalence of apoptosis were determined using the TUNEL assay (Figs. 2A–2C). Total cell numbers of blastocysts in the 2i treatment groups were significantly higher than in control group (Control: 57.32 ± 4.79 vs PD0325901: 59.70 ± 7.44 vs CHIR99021: 60.20 ± 5.20 vs 2i: 71.59 ± 7.90). However, GSK3 or 2i inhibition decreased the number of TUNEL-positive cells in blastocyst preparations (Control: 4.54 ± 0.41 vs PD0325901: 4.54 ± 0.62 vs CHIR99021: 2.83 ± 0.41 vs 2i: 2.04 ± 0.32). To confirm the regulation of apoptotic gene expression on 2i treatment, we carried out the qRT-PCR using control and 2i-treated blastocysts. Interestingly, anti-apoptotic gene *Bcl-2* mRNA levels was increased with 2i treatment. These results demonstrated that 2i treatment improve the blastocyst quality by affecting apoptosis and total cell number.

### Increasing of inner cell mass in MEK/GSK3 inhibition

**Figure 3:**
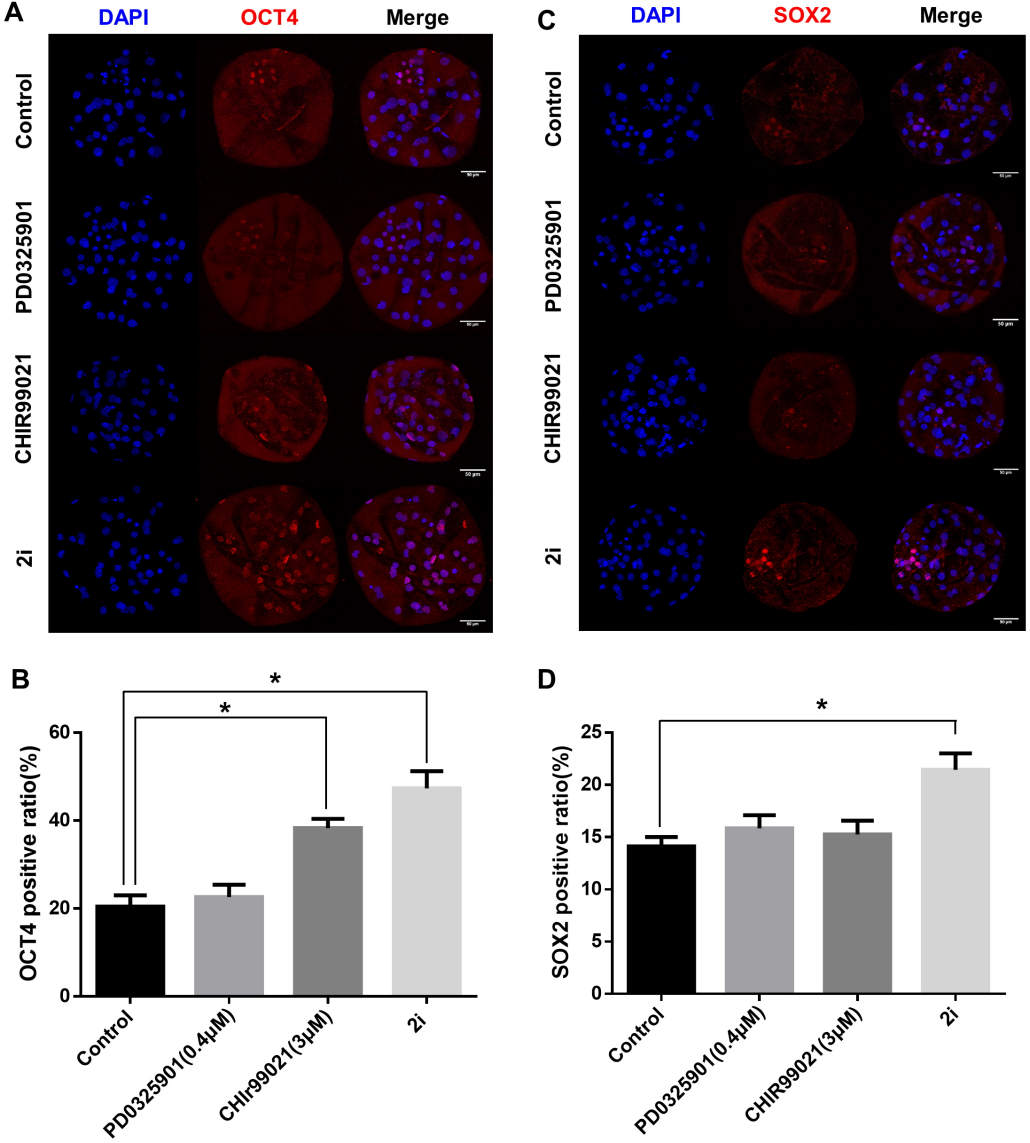
ICM marker protein expression after 2i treatment. (A) Immunofluorescence analysis for OCT4 (red) and Hoechst 33342 nuclear staining (blue) in inhibitor-treated blastocyst. (B) OCT4-positive cell ratio (%) in the nucleus. (C) Immunofluorescence analysis for SOX2 (red) and Hoechst 33342 nuclear staining (blue) in inhibitor-treated blastocyst, and (D) SOX2-positive ratio in the nucleus. Scale bars: 50 μm. *P < 0.01.

To determine the effect of 2i treatment on the ICM formation and expression levels of ICM marker proteins, we compared OCT4 and SOX2 expression in GSK3/MEK-inhibited, GSK3-inhibited, MEK-inhibited, and control blastocysts (Figs. 3A–3D). Inhibition of GSK3 or both MEK and GSK3 significantly increased the ratio of OCT4-positive cells (Control: 20.48 ± 2.55 vs PD0325901: 22.64 ± 2.80 vs CHIR99021: 38.33 ± 2.11 vs 2i: 47.37 ± 3.91). In addition, the SOX2-positive cell ratio in 2i-treated blastocysts were higher than in other groups (Control: 14.12 ± 0.90 vs PD0325901: 15.85 ± 1.23 vs CHIR99021: 15.27 ± 1.30 vs 2i: 21.46 ± 1.57). These results indicated that MEK/GSK3 inhibition 2i-treated blastocyst increase ICM cells in porcine early embryo development.

### Effect of MEK/GSK inhibition on the expression of on fate-related transcription factors and genes related to epigenetic modification

**Figure 4:**
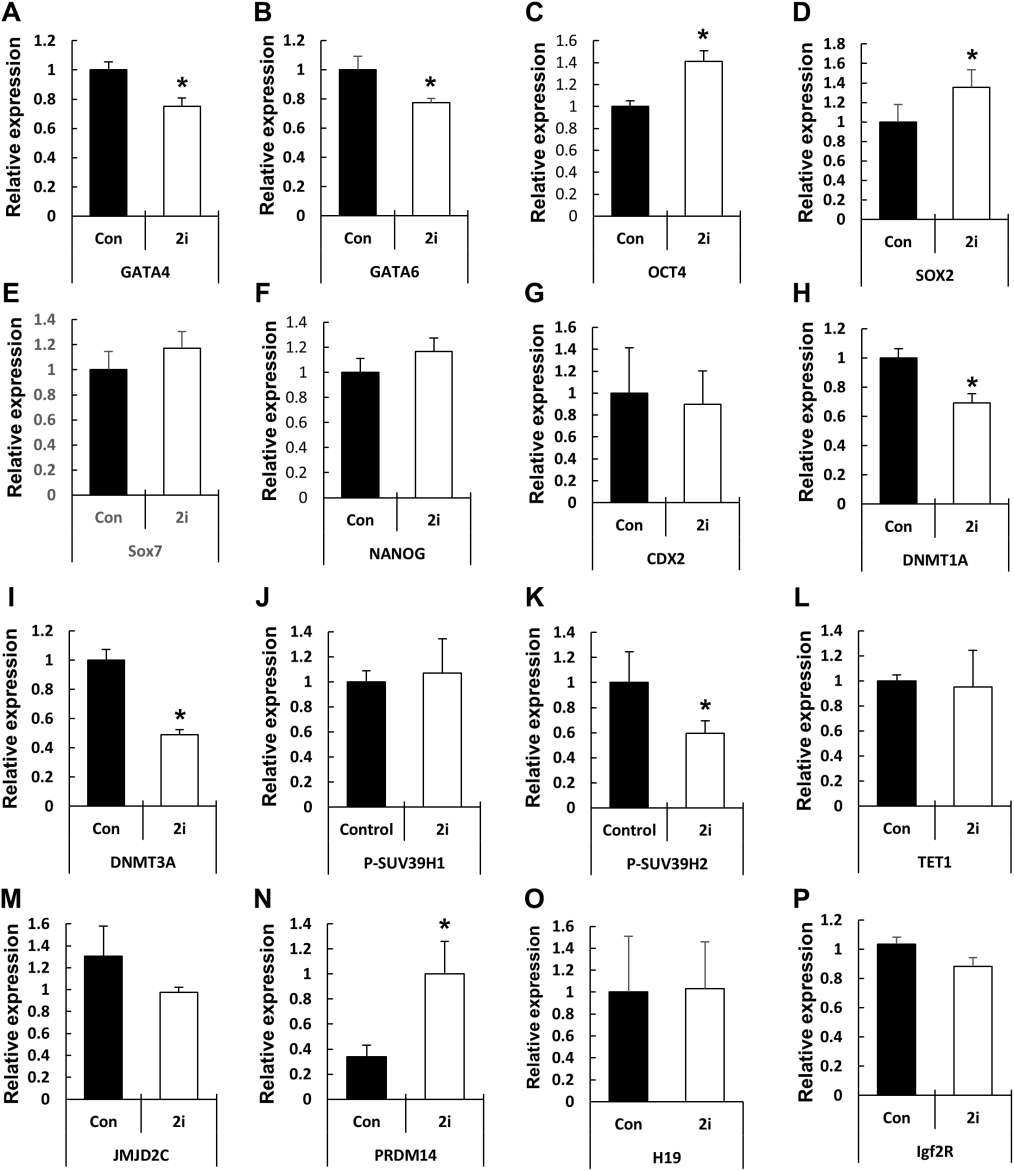
Changes in global gene expression patterns in 2i-treated blastocysts. Relative mRNA expression levels of ICM & TE differentiation (A–G), Histone modification & DNA methylation (H–N), and maternal imprinting genes (O and P). cDNA was isolated from day 7 blastocysts and used for qRT-PCR. Three experimental replicates were used for >20 embryos in each group. *P < 0.01.

Based on the phenotypic characterization of the 2i-treated porcine blastocysts, we speculated that MEK/GSK3 inhibition affects the expression of pluripotency-associated genes in blastocysts. Hence, we assessed the expression of various transcription factors involved in epiblast or trophoblast differentiation using qRT-PCR (Fig. 4). Compared to the control, *OCT4* expression was increased in the 2i-treated groups, whereas the expression of other pluripotent-related genes (*NANOG*, *SOX2*, and *CDX2*) showed no difference in expression. However, *GATA4*, a hypoblast differentiation-related gene, was significantly reduced in 2i treatment groups.

Next, we assessed the levels of genes related to epigenetic modifications. DNMT1A and DNMT3A mRNA levels were significantly reduced in 2i-treated blastocysts, whereas the expression of the DNA demethylase TET1 did not differ between the control and treated samples. Expression of the histone H3-K9 methyltransferase (such as SUV39H2) was reduced by 2i treatment, whereas the expression of other H3-K9 methyltransferases (such as *SUV29H1*) did not change. Furthermore, the expression levels of histone demethylases of the Jumanji domain 2 (*JMJD2*) family and *KDM4C* did not change with 2i treatment. Interestingly, the mRNA level of PR-domain-containing 14 (*PRDM 14*) gene, which is involved in the maintenance of pluripotency in mouse ESCs (Ma et al., 2011), was significantly higher in the 2i-treated groups.

## Discussion

In this study, we examined the effects of 2i treatment during porcine parthenogenetic embryogenesis, with a focus on blastocyst quality and expression of pluripotency markers. Previous studies on MEK/GSK3 inhibitor treatments focused on the establishment of ESCs or induced pluripotent stem cells (Li et al., 2009; Shi et al., 2008; Silva et al., 2008). In addition, the effect of 2i on embryogenesis of various mammalian species, including mouse (Nichols et al., 2009), rat (Buehr et al., 2008), and cattle (Harris, Huang & Oback, 2013), have been reported. Although the effects and the mechanism via which the 2i system regulates establishment of naïve stemness in rodent ESCs have been studied extensively, whether these mechanisms are conserved in the porcine embryo remains unclear. Germline transmission through ESCs has not been reported beyond the production of chimeras using porcine embryonic germ cells (Mueller et al., 1999; Piedrahita et al., 1998; Rui et al., 2004) or placental chimeras using an ESC-like cell line (Xue et al., 2016). Investigating signaling pathways involved in the differentiation and maintenance of stemness in the porcine embryo would be useful for the establishment of a porcine ESC line.

We observed that the 2i system improved blastocyst quality by reducing apoptosis and facilitating the expression of pluripotency marker genes in porcine embryos, similar to that observed in rodent embryos. We evaluated the effect of the 2i system on blastocyst quality during early porcine embryo development. Since inhibition of the MEK1/2 pathway induces apoptosis in epithelial cells (Ciuffreda et al., 2009), we assessed the extent of apoptosis and the expression patterns of related genes. Interestingly, MEK inhibition by PD0325901 did not change the level of apoptosis, whereas GSK3 inhibition or the concurrent inhibition of MEK and GSK3 effectively reduced apoptosis in treated embryos. These results indicate that MEK/GSK3 inhibition directly affects apoptosis in porcine embryos. Considering that the apoptotic pathway is generally induced by p53 after DNA damage (Williams & Schumacher, 2016) and that GSK3 is involved in caspase activation and apoptotic pathway induction (Watcharasit et al., 2002), we suggest that GSK3-mediated apoptosis and modulation of this pathway during porcine embryogenesis is important for the development and generation of porcine embryos. Although, we did not confirm p53 expression between control and 2i treatment group, because of absence of DNA damage induced condition. In previous work in radiation, p53-dependent apoptosis was reduced by CHIR99021 (Wang et al., 2015). Therefore, the relationship between 2i and p53-dependent apoptosis under DNA damage conditions during early embryo development requires further investigation.

Previous studies showed that the 2i system affects histone marker status in ESCs (Habibi et al., 2013; Marks et al., 2012). Since silencing of ICM-specific gene expression in TE cells (Rugg-Gunn et al., 2010) or TE marker gene expression in ICM (Yuan et al., 2009; Yeap, Hayashi & Surani, 2009) is important for the establishment of cell fate and embryo developments, the MEK/GSK3 inhibitor treatments can be expected to change the epigenetic modification levels and expression levels of relevant genes. H3K9me3 and H3K9ac levels changed in CHIR99021-treated and 2i (Habibi et al., 2013). In mammalian cells, JmjC domain-containing histone demethylase JMJD2C histone methyltransferase Suv39h2 regulated H3K9me3 (Garcia-Cao et al., 2004; Cloos et al., 2006). As expected, the mRNA level of the H3K9me3 methyltransferase SUV39H2 was reduced in 2i-treated embryos although no difference was observed in JMJD2C mRNA level (Fig. 4). These results demonstrated that MEK/GSK3 inhibition may regulate histone modification by regulating the levels of histone methyltransferases. The underlying mechanisms regulating histone methyltransferase expression via the MEK/GSK3 pathway are still unknown and require further investigation.

In addition to histone modification, remodeling of DNA methylation status is one of the major epigenetic modifications occurring during early mammalian embryogenesis (Rougier et al., 1998). Previous studies show that 2i reduced the expression of DNMT family proteins by increasing of PRDM 14 expression (Sim et al., 2017). Indeed, PRDM 14 regulates expression of the naive pluripotency-related genes via repress the expression of de novo DNA methyltransferases (such as DNMT3A, 3B, and 3L) (Yamaji et al., 2013). We didn’t confirm DNA methylation levels in 2i treated-embryos, but observed that DNMT1A and DNMT3A mRNA levels were reduced, whereas PRDM 14 mRNA level was increased by MEK/GSK3 treatments. These results indicating that passive DNA methylation status may change by DNA methyltransferase activity through MEK/GSK3 inhibition, and regulate pluripotency-related gene expression.

Our results demonstrate that MEK and GSK3 inhibition improved porcine blastocyst quality and affected expression of epigenetic modification and pluripotency-associated genes. We suggest that conservation of the MAPK and Wnt signaling pathways during differentiation and maintenance of porcine ICM and their inhibition can be useful for establishing naïve porcine ESCs.

## Supplemental Information

10.7717/peerj.5840/supp-1 Supplemental Information 1 Table S1. Primer sequences for qRT-PCR. List of primer sequences for qRT-PCR.

10.7717/peerj.5840/supp-2 Supplemental Information 2 Raw data. Raw data for the figures.

## Additional Information and Declarations

### Data Availability

The following information was supplied regarding data availability: The raw data are provided in a Supplemental File.
