# Supplementary material for: Inhibition of MEK1/2 and GSK3 (2i system) affects blastocyst quality and early differentiation of porcine parthenotes
Source: PeerJ. 2019 Jan 7;6:e5840. doi: 10.7717/peerj.5840 (PMC6327883; doi:10.7717/peerj.5840)
Supplement: Supplemental Information 1 — List of primer sequences for qRT-PCR. [file peerj-07-5840-s001.docx]

**S1 Table. Primer sequences for qRT-PCR.**

| **Gene** | **Sequence (5’– 3’)** | **Size (bp)** |
| --- | --- | --- |
| **GAPDH** | **F: GGGCATGAACCATGAGAAGT**  **R: AAGCAGGGATGATGTTCTGG** | **230** |
| **GATA4** | **F: TCAAACCAGAAAACGGAAGC**  **R: GGCCAGACATGGCACTAACT** | **230** |
| **GATA6** | **F:** **CTGTCCCCATGACTCCAACT**  **R:** **ATGTACAGCCCGTCTTGACC** | **178** |
| **OCT4** | **F: CGTGGAGACTTTGCAGCCTGA**  **R: GGCGATGTAAGTGATCTGCTG** | **519** |
| **SOX2** | **F: GCCCTGCAGTACAACTCCAT**  **R: GCTGATCATGTCCCGTAGGT** | **216** |
| **SOX7** | **F:** **CAAGGACGAGAGGAAACGTC**  **R:** **GTTGGGGTAGTCCTGCATGT** | **166** |
| **NANOG** | **F: AAGTACCTCAGCCTCCAGAC**  **R: GTGCTGAGCCCTTCTGAATC** | **232** |
| **CDX2** | **F: AGCCAAGTGAAAACCAGGAC**  **R: TGCGGTTCTGAAACCAGATT** | **178** |
| **DNMT1A** | **F: GTGAGGACATGCAGCTTTCA**  **R: AACTTGTTGTCCTCCGTTGG** | **211** |
| **DNMT3A** | **F: CTGAGAAGCCCAAGGTCAAG**  **R: CAGCAGATGGTGCAGTAGGA** | **238** |
| **SUV39H1** | **F: CTGCACAAGTTCGCCTACAA**  **R: TCTTGCGGATCTTCTCCAGT** | **202** |
| **SUV39H2** | **F: GACTTAGAGGGCCCACCTTC**  **R: TGCCAAAAGAACTCCAGCTT** | **153** |
| **Tet1** | **F: TGGGTTTACAATGGCTCTCC**  **R: TCTTGCTTTGGGACTTGCTT** | **192** |
| **JMJD2C** | **F: GAGGACGAGGAAGTGTCAGC**  **R: AGCCTCGGTTTTGATTTCCT** | **246** |
| **PRDM14** | **F: CTTCACGTCCATGAGAAGCA**  **R: GGGACGCAAAAGATTTACCA** | **232** |
| **H19** | **F: CTCAAACGACAAGAGATGGT**  **R: AGTGTAGTGGCTCCAGAATG** | **122** |
| **IGF2R** | **F: AGGTCTCACCTCTTCAGGTT**  **R: CTGTGCAAATTAAGGCTTCT** | **120** |

**(F: forward. R: reverse)**
